# The Timing of Utterance Planning in Task-Oriented Dialogue: Evidence from a Novel List-Completion Paradigm

**DOI:** 10.3389/fpsyg.2016.01858

**Published:** 2016-12-01

**Authors:** Mathias Barthel, Sebastian Sauppe, Stephen C. Levinson, Antje S. Meyer

**Affiliations:** ^1^Language and Cognition Department, Max Planck Institute for PsycholinguisticsNijmegen, Netherlands; ^2^Department of Comparative Linguistics, University of ZurichZurich, Switzerland; ^3^Donders Institute for Brain, Cognition and Behaviour, Radboud UniversityNijmegen, Netherlands; ^4^Psychology of Language Department, Max Planck Institute for PsycholinguisticsNijmegen, Netherlands

**Keywords:** timing of turn-taking, task-oriented dialogue, prediction, production, planning, eye-movements

## Abstract

In conversation, interlocutors rarely leave long gaps between turns, suggesting that next speakers begin to plan their turns while listening to the previous speaker. The present experiment used analyses of speech onset latencies and eye-movements in a task-oriented dialogue paradigm to investigate when speakers start planning their responses. German speakers heard a confederate describe sets of objects in utterances that either ended in a noun [e.g., *Ich habe eine Tür und ein Fahrrad* (“I have a door and a bicycle”)] or a verb form [e.g., *Ich habe eine Tür und ein Fahrrad besorgt* (“I have gotten a door and a bicycle”)], while the presence or absence of the final verb either was or was not predictable from the preceding sentence structure. In response, participants had to name any unnamed objects they could see in their own displays with utterances such as *Ich habe ein Ei* (“I have an egg”). The results show that speakers begin to plan their turns as soon as sufficient information is available to do so, irrespective of further incoming words.

## 1. Introduction

Most psycholinguistic studies are directed at detailed processes in either comprehension or production, testing single participants in isolation. Yet, interactive language use involves both, not only in rapid succession but also in partial overlap. In conversation, the predominant form of language use, interlocutors fluently engage in switching of roles, taking turns at talking with only about 200 ms between turns on average (Sacks et al., 1974; de Ruiter et al., 2006; Stivers et al., 2009; Heldner and Edlund, 2010; Levinson and Torreira, 2015; Levinson, 2016). One factor that maintains this pace is that markedly delayed turns carry a special semiotics, presaging disagreement or non-compliance with what was said before (Levinson, 1987; Roberts et al., 2011; Roberts and Francis, 2013; Kendrick and Torreira, 2014; Bögels et al., 2015a).

Given the known latencies involved in speech production of 600 ms or more for a single word in picture naming tasks (Levelt, 1989; Jescheniak et al., 2003; Indefrey and Levelt, 2004; Strijkers and Costa, 2011) and over 1500 ms for simple sentences in scene description tasks (Griffin and Bock, 2000; Schnur et al., 2006), this brief interval between turns will often not allow speakers sufficient time to plan and initiate a response (Griffin, 2003). It therefore seems likely that next speakers prepare their response partly while the incoming turn is still unfolding. A model of turn-taking based on these observations has recently been formulated by Levinson and Torreira (2015). In this model, the listener as next speaker tries to anticipate the action carried out with the incoming turn (e.g., a request) early during the turn and begins to conceptualize and formulate a response as soon as the action becomes clear. Parallel to content planning and formulation, the next speaker (predictively) parses the input for possible points of syntactic closure and other cues to turn completion, while a formulated response may be temporarily held in a buffer. As the incoming turn is about to end, the next speaker prepares the articulators and initiates response. Hence, the model accounts for short gaps between turns by assuming that content planning starts as early as possible, comprehension continues in parallel with response preparation, and articulation can be launched from a prepared formulation when transition becomes relevant. Such parallel processing should be cognitively demanding, since speaking and listening can interfere with one another and are known to take up processing resources (Schriefers et al., 1990; Kemper et al., 2003; Kubose et al., 2006; Boiteau et al., 2014; Sjerps and Meyer, 2015) and are partly supported by the same neurological system (Hagoort et al., 1999; Menenti et al., 2011; Kempen et al., 2012; Segaert et al., 2012). Thus, speakers face the task of producing a response under time pressure while keeping capacity demands and interference between comprehension and production within reasonable bounds. In their parallel processing model, Pickering and Garrod (2013) propose that fluent turn-transitions are made possible by forward modeling of the incoming speech signal with the help of the addressee's own production system (cf. also Garrod and Pickering, 2015). In this account, the addressee is taken to covertly imitate the production of the incoming turn based on the input that has already been transmitted and thereby anticipate the content and timing of the incoming turn so as to be able to prepare a response in a timely fashion. Irrespective of whether or not the production system is used to imitate the incoming turn, early anticipation of the incoming turn's message and intended action would be a necessary pre-requisite for early response preparation.

Another task of next speakers is to detect when the incoming turn comes to an end and speaker transition becomes relevant. Sacks et al. (1974) hypothesized that listeners predict the end points of the incoming turns using syntactic and prosodic cues to turn closure (see also Ford and Thompson, 1996). They suggested that the projection of upcoming turn-completion points was essential for the close timing observed in conversation. Using experimental evidence for turn end estimation, de Ruiter et al. (2006) claimed that lexico-syntactic cues are essential for accurate projection of turn completion points, which, in their view, is a necessary pre-requisite for response planning (see also Riest et al., 2015). Based on this assumption, de Ruiter et al. (2006) hypothesized that response turns could only be planned when the end point of the incoming turn can be accurately projected, meaning that a response could not be planned without knowing the duration of the rest of the incoming turn (Projection-Dependent Hypothesis). Contrary to this hypothesis, based on their quantitative analysis of conversational speech corpora, Heldner and Edlund (2010) claimed that at least about 40% of turn transitions could be explained without the assumption of turn end projection.

The alternative to the hypothesized projection-dependent planning is that speakers begin to plan their utterance without knowing precisely when the current turn will end and, if necessary, postpone articulation until they detect a turn-completion point, as described in the model by Levinson and Torreira (2015) (Projection-Independent Hypothesis). On this account, the exact syntactic structure and words of the incoming turn do not need to be predicted for response planning to begin. Instead, merely the turn's message or intentions need to be known or anticipated, using the many contextual cues available from the organization of conversational sequences (Schegloff, 2007), common ground (Clark, 1996), or general knowledge about the speaker, the environment, and the world. As soon as speakers can anticipate the interlocutor's intention they can allocate some of their computational resources to their own planning processes (Gisladottir et al., 2015). Thus, if the interlocutor's message can be recognized or anticipated early during their turn, response planning, i.e., conceptualization and formulation, can begin early as well.

The present study tests the hypotheses that (a) response planning starts as early as the incoming turn's message can be anticipated, and (b) that the onset of response planning depends on an accurate projection of the incoming turn's completion point.

A small number of previous studies have set out to investigate when response planning in dialogue starts and whether a projection of the turn end is necessary for response planning to begin. Their results are not fully consistent. Magyari et al. (under review) addressed both of these questions. They investigated whether participants would start planning a response earlier during a question if the answer could be known early on vs. only at the last word of the question. Visual displays were used that contained a tiger and a rabbit, each with or without further objects attached to them. Participants heard questions of the format *Which animal has object X and object Y?*, with the answer being available either already before the beginning of the question (early condition, with only one animal with objects) or only with the last object (late condition, with both animals with objects and only the last object being different between animals). Answers were faster in the early condition than in the late condition, suggesting that response planning was not delayed until the end of the question. The second question that Magyari et al. examined was whether participants anticipated when exactly the question would end so as to be able to time their answer accurately to the end of the question. The lengths of the names of the objects were manipulated so that the length of the question could either be accurately projected (congruent condition, with the last objects having equally long names) or not (incongruent condition, with the last object having names of different lengths). No main effect of congruence was found, giving no support to the hypothesis that an accurate projection of a turn's completion point is necessary to plan a response.

Bögels et al. (2015b) used EEG measurements to track the time course of comprehension and production processes in a quiz-like situation. Participants heard quiz questions to which the answer could be known either mid-sentence or only at the very end of the question, such as *Which character, also called 007 appears in the famous movies?* (early condition) and *Which character from the famous movies is also called 007?* (late condition). At both the early and the late time points, they found significant positive deflections after 500 ms in questions containing the critical word (giving away the question) as compared to the respective questions that did not contain the critical word in that position. In a control experiment in which participants did not have to answer the questions but remember them, this effect was substantially reduced. The authors concluded that speech planning began as soon as all information needed to provide an answer was available.

Boiteau et al. (2014) investigated the cognitive load arising in different phases of a conversation using a dual-task paradigm. Participants continuously tracked a point on the screen with their computer mouse while freely talking to either a confederate or a friend. Tracking performance was worse during speaking than during listening and began to decline already about 250–450 ms before the end of a listening turn. The authors concluded that speakers already began to plan their utterance while still listening to their interlocutor.

Sjerps and Meyer (2015) also investigated cognitive load during the temporal overlap between listening and planning using a dual-task paradigm. Participants continuously tapped their fingers in a predefined order while listening to a recorded description of a row of pictures and subsequently described a second row of pictures before a time-out signal. Whether the recording referred to the top or bottom row varied randomly from trial to trial, but as soon as the participants heard the first noun, they knew which row was being described and could, in principle, prepare for the description of the other row. Nonetheless, both participants' eye movements and tapping performance indicated that planning began quite late, only shortly before, or at the very end of the recorded turn. These results do not support the view that speakers begin to plan their utterance as soon as they have understood the message of the incoming turn. Rather, the authors suggest, response planning began much later, perhaps to avoid interference between listening and planning. However, there are a number of reasons that call for caution when generalizing the observed timing of the relevant processes to everyday conversation. First, as there was no interlocutor present, the validity of generalizing the results to live interaction is unknown. Second, all turns, incoming and response, had the same syntactic structure and length. Consequently, the timing of the ends of incoming turns was highly predictable, and the beginnings of response turns could easily be held in working memory. Third, only forty objects were used in the item displays and they were reused twenty-one times, potentially influencing participants' planning strategies. Finally, even though participants only prioritized planning over listening toward the end of the recorded trial, they may have planned the beginnings of their responses already during the recorded utterance, looking at the target object for only a short period and then returning their gaze for comprehension. As the incoming turns were very long, such early looks may be distributed across the incoming turn and are therefore difficult to detect.

To summarize, the studies reviewed here came to different conclusions when investigating when next speakers begin to plan a response and whether they rely on projectable turn-completion points to initiate response planning. Two possible hypotheses about the timing of planning are proposed: Next speakers prioritize planning as soon as they have understood or can anticipate the message of the incoming turn, as put forward by Bögels et al. (2015b) and incorporated in the model by Levinson and Torreira (2015) (Early Planning Hypothesis), or only when the incoming turn is coming to completion, as postulated by Sjerps and Meyer (2015) (Late Planning Hypothesis). Similarly, two possible hypotheses about the necessity of precise projection of the incoming turn's completion point are proposed: Next speakers depend on a projection of the incoming turn's end, as proposed by de Ruiter et al. (2006) (Projection-Dependent Hypothesis) or they can start planning their response without an accurate projection, as modeled by Levinson and Torreira (2015) (Projection-Independent Hypothesis). The experiment described in the following was designed to evaluate these hypotheses.

## 2. The current experiment

The study presented here made use of a novel task-oriented dialogue paradigm, the list-completion paradigm. A female confederate and a participant jointly completed a task while sitting in separate sound proof booths in front of monitors and talking to one another via microphones and headphones without visual contact. Unbeknownst to the participant, most of the critical utterances of the confederate were pre-recorded prior to the experiment and played back by the confederate at the relevant moments during the experiment. In this way, the participant heard the utterances as being produced live and spontaneously by the confederate, fitting the conversational flow. A similar approach of combining live and pre-recorded playback modes was taken by Bögels et al. (2014).

**Figure 1:**
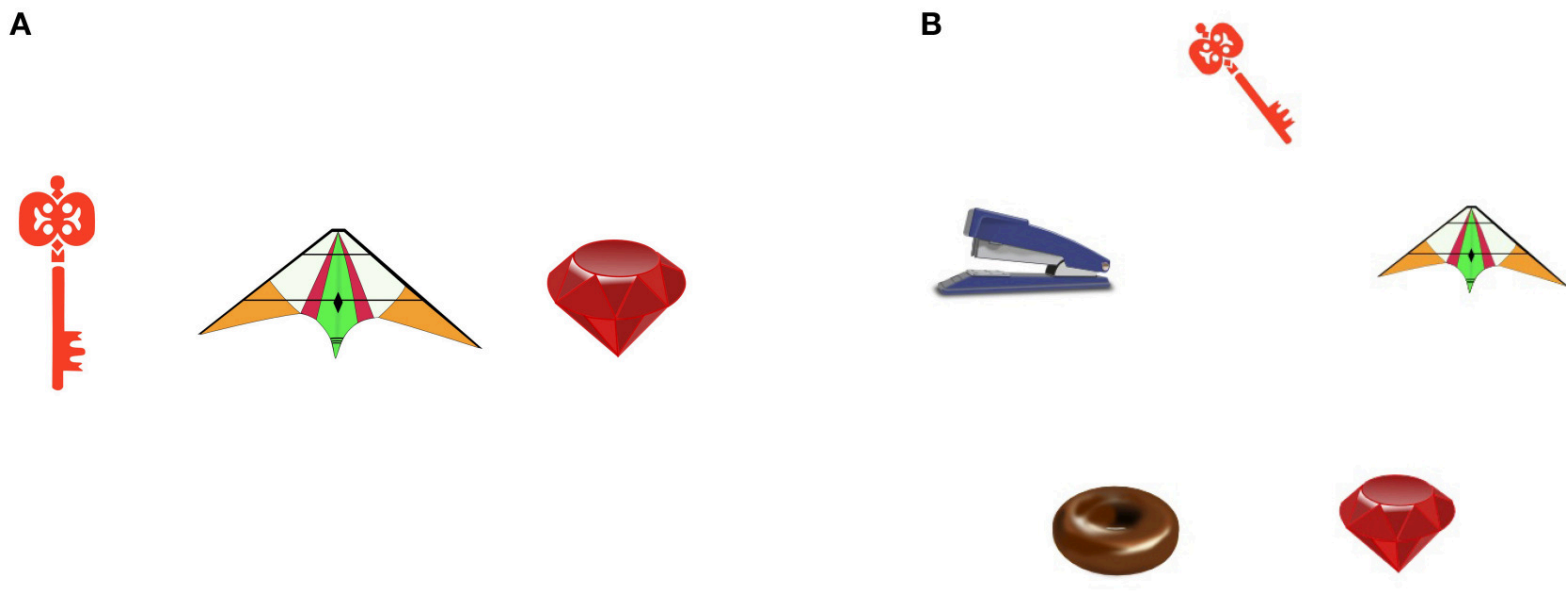
**Example item displays**. **(A)** confederate display. **(B)** participant display.

On their screens, participants saw stimuli with differing numbers of objects (cf. Figure 1 for an example). The confederate named the objects on her screen and the participant subsequently named all additional objects displayed on their screen. All speech was audio recorded. Moreover, participants' eye-movements were recorded. It was assumed that participants' gaze would follow the objects that are named by the confederate while comprehending the object names, and would move on to the objects that had to be named while planning the response turn (Just and Carpenter, 1980; Griffin and Bock, 2000; Tanenhaus et al., 2000; Griffin, 2001; Altmann and Kamide, 2007; Huettig et al., 2011).

The experiment was conducted in German. The confederate's critical turns appeared in four conditions, differing in syntactic structure. The four conditions formed a 2 × 2 design (Table 1). The first binary factor was projectability of the turn ending (±Pend), i.e., whether it was projectable or not how a turn would end (either in the last object name of the list or a turn final verb). −Pend conditions contained the verb *habe* (“have”) in second position. In this position, *habe* was ambiguous as to whether it was a main verb or an auxiliary, which in turn would require a main verb in sentence-final position, in this case *besorgt* (“gotten”). Both meanings of *habe* were used in the experiment. Therefore, sentences in the two −Pend conditions did not allow a precise projection of when they would end. Sentences in the +Pend conditions either contained the main verb *sehe* (“see”) or the modal verb *kann* (“can”), which required another verb in sentence-final position, in this case *besorgen* (“get”). Therefore, sentences in the two +Pend conditions allowed for a precise projection of their completion point. The second factor was the presence or absence of a sentence-final verb (±Vend). Sentences in the −Vend condition ended right after the object list, whereas sentences in the +Vend condition ended after a sentence-final verb. While the number of objects named by the confederate varied from trial to trial, the last object noun was always preceded by *und* (“and”) or, in the case of items with only one object being named, *nur* (“only”), providing a clear lexical cue to the end of the object list.

**Table 1:** **Example sentences of the four conditions used in the experiment**.

**Condition**		**Projectable ending or not**	
		**−Pend**	**+Pend**
**Verb in final position or not**	−**Vend**	Ich habe einen Schlüssel, einen Lenkdrachen und einen Rubin.	Ich sehe einen Schlüssel, einen Lenkdrachen und einen Rubin.
	+**Vend**	Ich habe einen Schlüssel, einen Lenkdrachen und einen Rubin besorgt.	Ich kann einen Schlüssel, einen Lenkdrachen und einen Rubin besorgen.

“I have/have gotten/see/can get a key, a kite, and a ruby.”

The timing of participants' looks for planning and their response latencies were measured. For both measures, the contrasted hypotheses make different predictions. According to the Early Planning Hypothesis, participants should start planning as soon as they recognize the last object of the incoming list and should use the duration of a turn final verb to start planning their response. According to the Late Planning Hypothesis, however, participants should start planning only when the turn-completion point is reached and would not gain extra planning time in turns with sentence-final verbs.

Eye-movements were analyzed using growth curve modeling (Mirman et al., 2008; 2014), a variety of mixed effects regression that makes use of polynomial time terms as predictors to model differences in fixation likelihoods. Linear, quadratic and cubic time terms were included. The linear time term (Time) models the overall increase in fixations over the time course of a trial. The quadratic time term (Time^2^) models the steepness of the curve, i.e., how “U-shaped” it is. The cubic time term (Time^3^) describes whether fixations increase earlier or later (“S-shaped” curve).

The Early Planning Hypothesis predicts no difference between the two conditions in the moment in time at which participants shift their gaze for planning, measured from the beginning of the turn. In terms of the analyses applied, this is a prediction of null effects of Time^3^ × ±Vend. It further predicts a main effect of ±Vend in response latencies, with faster responses after turns with a final verb (+Vend) than after turns without a final verb (−Vend), because participants should gain extra planning time at the end of the incoming turn if it ends in a verb. The Late Planning Hypothesis predicts that participants shift their gaze for planning later in turns with a turn-final verb (+Vend) than in turns without (−Vend), which would manifest as an effect of Time^3^ × ±Vend. It further predicts a null effect of ±Vend in response latencies because no extra time for planning should be gained in turns with sentence-final verbs.

According to the Projection-Dependent Hypothesis, participants should start planning as soon as they recognize the last object of the incoming list after turns with projectable endings (+Pend), whereas after turns with unprojectable endings (−Pend) they should start planning only upon recognizing whether a turn-final verb form follows the object list or not (i.e., only when they can project when exactly the turn will come to an end). According to the Projection-Independent Hypothesis however, participants should start planning as soon as they recognized the last object of the incoming list in all conditions.

Consequently, the Projection-Dependent Hypothesis predicts participants to shift their gaze for planning earlier (measured from the beginning of the turn) in turns with projectable endings (+Pend) than in turns with non-projectable endings (−Pend), which would manifest as an effect of Time^3^ × ±Pend. It further predicts a main effect of ±Pend on response latencies, with faster responses after projectable turns than after unprojectable turns, since participants could start planning longer before the end of the turn when its completion point was projectable. The Projection-Independent Hypothesis predicts no difference in the moment in time at which participants shift their gaze for planning, which would manifest as null effects of Time^3^ × ±Pend. It further predicts a null effect of ±Pend in response latencies.

The timing pattern of response planning as described by Levinson and Torreira (2015) results in overlap of comprehension and production processes at the junction of turns, where planning already begins while the incoming turn is not yet complete and comprehension is still going on, as predicted during turns with sentence-final verbs in the present study. Since several of the studies reviewed above found interference effects of incoming speech on response planning (Schriefers et al., 1990; Kemper et al., 2003; Boiteau et al., 2014; Bögels et al., 2015b), planning during the turn-final verbs would be hypothesized to be less efficient than planning during silence. This difference in efficiency should manifest as an effect of Time^2^ × ±Vend, with proportions of looks for planning increasing more slowly in turns with a final verb than in turns without a final verb. Furthermore, this difference could be modulated by the projectability of the turn-final verb, since incoming words might be less detrimental to response planning when they can be projected than when they cannot. This influence of projectability of final verbs should manifest as an effect of Time^2^ × ±Pend in turns with a final verb. Both hypotheses about the influence of verb finality and projectability on the efficiency of response planning will be tested in the present study.

## 3. Materials and methods

### 3.1. Participants

Forty-eight German native speakers (30 female) were tested as paid participants at Heinrich-Heine University, Düsseldorf, Germany. All participants reported to have normal or corrected-to-normal vision and normal hearing abilities. Eight participants stated in a questionnaire filled in after the experiment that they noticed the presence of pre-recorded materials. These participants were excluded from the analyses. Remaining participants had a mean age of 26.3 years (SD = 7.6).

### 3.2. Apparatus

The participant and the confederate were seated in separate sound proof booths approximately 60 cm away from 21″ computer screens. They were unable to see each other and could only communicate via microphones and headphones. The participants' eye-movements were recorded with an SMI RED-m remote eye-tracker (120 Hz sampling rate).

### 3.3. Visual stimuli

Four-hundred and sixty-eight pictures of objects were used in the experiment. The pictures were sourced online and are under creative commons license. They were selected to be easy to recognize and name. All pictures, with the exception of twenty pictures used in practice trials, showed inanimate objects.

One-hundred and seventeen pairs of item displays (participant displays and corresponding confederate displays) that showed a differing number of objects drawn from the pool of object pictures were used as visual stimuli (see Figure 1 for an example). The participant displays showed between three and five objects. These objects included all objects shown on the corresponding confederate display and zero, one, two, or three further objects. In participant displays that showed three objects, the objects formed an equilateral triangle, when showing four objects, the objects formed a square, when showing five objects, the objects formed an equilateral pentagon. Objects on the displays filled approximately two degrees of visual angle. They had equal distances of about four centimeters to their neighbors, irrespective of the arrangement they were presented in on the display. That means that to see the individual objects sharply, participants had to move their eyes to focus on them. The most common names of the objects of a display did not start with the same phoneme. Names of objects that were named by the participants had a midrange frequency. Names of objects that were named by the confederate were sampled from wider frequency ranges (based on the German Wortschatz Corpus, Department of Computer Science, Universität Leipzig, 2016).

Ninety-six displays were critical test displays, with 32 displays each showing three, four, or five objects on the participant display. The confederate displays showed between zero and five objects, so that 24 participant displays showed no more objects than the corresponding confederate display, 24 participant displays showed one more object, 24 participant displays showed two more objects, and 24 participant displays showed three more objects.

In the test phase, nine pairs of displays were used as displays for live items (see Auditory stimuli below). Three participant displays in this group of items showed three objects, three showed four objects, and three showed five objects. The confederate displays in this group of items showed between zero and four objects, so that three of the corresponding participant displays showed one more object than the confederate display, three showed two more objects, and three showed three more objects.

The experiment was preceded by a practice phase using twelve display pairs.

### 3.4. Auditory stimuli

Sentences accompanying ninety-six of the visual displays were pre-recorded in the same sound protected booth that was used for the experiment, using a unidirectional Sennheiser ME64 microphone attached to a digital flash recorder. Each sentence was recorded in the four conditions exemplified in Table 1. When the sentence contained two or more object nouns, the last noun was preceded by *und* (“and”). When it contained only one object noun it was preceded by *nur* (“only”). When it did not contain any object nouns, the object list was replaced by *nichts* (“nothing”), as in *Ich habe nichts (besorgt)* [“I have (gotten) nothing”].

Due to the structures of the sentences, their duration was confounded with the experimental conditions because the turn-final verbs in the +Vend condition were approximately 600 ms long, while there was no word following the list of objects in sentences in the −Vend conditions. Therefore, sentence length will be controlled for in the statistical analyses.

The pauses between object nouns were adjusted for the different versions of each sentence with Praat (Boersma and Weenink, 2015) to have random lengths between 400 and 600 ms, imitating the gaps in the original recordings. None of the list contours of the pre-recorded stimuli used in the experiment contained downsteps on non-final items (cf. Selting, 2007) and all sentences ended in a low boundary tone (cf. von Essen, 1956).

Sentences accompanying nine visual displays were not pre-recorded but produced live by the confederate during the experiment (+Live items). The sentences accompanying the twelve practice trials were also produced live. These sentences were produced so as to sound similar to the pre-recorded sentences, using the same verbs and syntactic structures that were used in the pre-recorded sentences. They were included to test for the comparability of participant's response timings after live and pre-recorded stimuli (±Live) in order to validate the assumption that responses after pre-recorded stimuli were given naturally.

### 3.5. Items and design

A participant display in combination with the accompanying sentence constituted an experimental item. In two thirds of the items in which the confederate named at least one object, the objects were arranged in clockwise order as they were named, starting at the top of the display. In one third of the items, including all +Live items, other arrangements were used, so as to ensure that the participants had to listen attentively and search for the items mentioned by the participant, rather than scanning the objects in the same order on all trials. Analyses controlled for this order-of-objects variable.

Four lists were constructed, with each sentence and the accompanying display appearing once per list and appearing in a different condition in each of the lists. In each list the same number of items appeared in each condition. Each participant was assigned to one of the lists. The order of the items in a list was randomized for every participant.

### 3.6. Procedure

#### Familiarization and instructions

Participants were invited to the lab to take part in a dialogue experiment. They were the first to enter the lab and told that the other participant of the study would arrive in a few minutes. In the meantime, participants were given a picture booklet containing all pictures used in the experiment and asked to name them. In 1.4% of all cases and in 0.9% of the cases involving pictures to be named by participants, the pictures were not recognized or labeled by participants, and a name was provided by the experimenter. The experimenter noted down participants' responses. The familiarization phase was audio-recorded.

After the familiarization phase, the confederate arrived and was introduced as a second participant. Participant and confederate were informed that they would be seated in separate booths and talk to each other via headphones and microphones to play the following game. They would see a number of displays on their respective screens, showing things they could get. The confederate was to tell the participant which things she has got already, so that the participant could tell the confederate what *further* objects (s)he could get. Participants were not instructed to use any particular utterance format.

The confederate was instructed to try to remember which objects she had seen and which names she had heard. This served as a cover task to distract participants from the aim of the study. Participants were told that their eye-movements would be recorded in order to study looking behavior when searching for objects on a screen whose names were heard. After instructions were given, the eye-tracker was calibrated. Calibration was repeated three times during the experiment.

#### Test phase

Before the beginning of the test phase, participants completed twelve practice trials, where instructions were repeated if necessary. During the test phase, all communication between the participants and the confederate was live, except for ninety-six pre-recorded sentences accompanying the critical displays. The confederate started the presentation of the stimulus displays and the corresponding pre-recorded utterances by button press so as to make them fit naturally into the conversation. Similarly, she produced the sentences accompanying the nine +Live items naturally in the flow of the conversation.

Participants were asked to look at a fixation cross that was presented in the center of the display at the beginning of each trial, which triggered the presentation of the item displays. After a preview of 600 to 1000 ms, the stimulus sentence began. Preview times varied randomly between items.

The experiment took about 30 minutes. After the experiment, participants were asked in a computerized questionnaire whether they had noticed the presence of pre-recorded speech. The entire test session took about 70 minutes, including familiarization, test phase and questionnaire.

## 4. Results

Fixation likelihoods and response latencies were the dependent variables. Statistical analyses are based on linear mixed effects regression models fitted in R (R Core Team, 2014) using the package lme4 (Bates et al., 2015). The maximal random effects structure justified by design was used for all models (Barr, 2013; Barr et al., 2013). Control variables were not included in the random effects structure. All categorical variables were deviation coded (−0.5 and 0.5). Statistical significance was assessed with *F*-tests with Kenward-Roger approximations of degrees of freedom (Kenward and Roger, 1997; Fox and Weisberg, 2011; Halekoh and Højsgaard, 2014). We report all data exclusions, all manipulations, and all measures in the study.

### 4.1. Response timing

Response latencies for the 3980 critical turn transitions were measured manually with Praat (Boersma and Weenink, 2015). They were operationalized as time intervals between the end of the incoming turn and the beginning of the response turn, ignoring any non-speech sounds like audible in-breaths. Trials were coded with respect to the verb structure produced by the participants in the critical responses. When participants used the same verbs as in one of the four stimuli conditions (*habe, habe besorgt, sehe, kann besorgen*), trials were coded parallel to the conditions (±Pend; ±Vend). All other response structures were coded as “other.” Response structure was used as a control variable in the mixed effects regression to control for any differences in response time that are due to the structure of the response turn rather than the structure of the incoming turn. Forty-nine percent of response structures were congruent to the structure of the corresponding confederate turn. Therefore, structural congruency (henceforth ±Priming) was included as a control variable in the analyses to control for any priming effects on the dependent variables, since responses repeating the structure of the previous turn might have been produced faster.

Twenty-four trials were excluded either because participants did not only name the correct objects or due to technical failure. Response latencies ranged from -211 ms to 3132 ms (M_*RL*_ = 806 ms, SD_*RL*_ = 370 ms, N_*RL*_ = 3956, Table 2).

**Table 2:** **Response latencies by condition**.

**Condition**			**Mean (SE) in ms**
**Format**	**Pend**	**Vend**	
*habe*	−	−	842 (11)
*habe …besorgt*	−	+	749 (11)
*sehe*	+	−	867 (12)
*kann …besorgen*	+	+	761 (11)

For the statistical analyses, thirty-five data-points (1%) were removed from the data set since they were outliers of more than three standard deviations of the mean response latency of the respective participant that produced the data-point.

Turns in conditions with a turn-final verb (+Vend) were longer than corresponding turns in conditions without a turn-final verb (−Vend) due to the presence or absence of the verb. Turn length might have affected response production processes. Magyari et al. (under review) found participants to answer questions faster the longer the question, irrespective of the content of the question or when the answer could be known. 'Magyari et al. propose that next speakers' level of preparedness to speak increases as the likelihood that the incoming turn will come to an end increases as the turn unfolds. Therefore, the duration of the critical turns was included as a control variable in the analyses.

To test whether the response latencies after pre-recorded items were the same as after live items, a model was fitted with playback mode as predictor (±Live). The duration of the confederate turns, as well as ±Priming, and a binary order-of-objects variable were included as control variables. Playback mode did not influence response latencies in this model [β = 22, *SE* = 41, *F*_(1, 15)_ = 0.30, *p* = 0.58]. Hence, data gained with pre-recorded items were regarded as ecologically valid and the following analyses are restricted to these items.

To evaluate the contrasting hypotheses formulated above, i.e., the Early vs. the Late Planning Hypothesis and the Projection-Dependent vs. -Independent Hypothesis, respectively a model was fitted to predict response latencies after pre-recorded turns, with ±Vend and ±Pend as well as their interaction and the duration of the confederate turn as predictors. The syntactic structure of the responses, as well as ±Priming were included as control variables. The model revealed a significant main effect of ±Vend [β = −92, *SE* = 15, *F*_(1, 46)_ = 42.62, *p* <   0.001], i.e., participants responded faster after turns that contained a final verb than after turns that did not end in a verb. Projectability did not significantly influence response latencies, nor did the interaction of projectability and verb position, meaning that response latencies were not modulated by the projectibility of a turn's ending. Response latencies were significantly shorter with increasing durations of the incoming turns [β = −17, *SE* = 6.40, *F*_(1, 76)_ = 6.50, *p* < 0.05]. This supports Magyari et al.'s (under review) finding that readiness to speak increases with increasing turn length. See Table 3 for a model summary. The analysis was repeated with the duration measured from the end of the confederate's turn to the beginning of the first object noun of the participant's turn (instead of the turn's beginning) as the dependent variable, yielding the same general pattern of results. In sum, the results support the Early Planning Hypothesis and the Projection Independent Hypothesis.

**Table 3:** **Response timing model and *F*-tests**.

	**Estimate**	**SE**	***t***	***F*(Df,Df.res)**	**Sig**.
(Intercept)	851.205	36.8	23.121		
Vend_yes	−92.002	14.9	−6.172	42.62(1, 46)	^***^
Pend_yes	23.598	16.5	1.430	2.00(1, 60)	n.s.
Vend_structure_yes	−11.954	15.7	−0.760	0.52(1, 727)	n.s.
Pend_structure_yes	0.089	16.6	0.005	0.00(1, 606)	n.s.
priming_yes	−32.381	12.9	−2.494	5.42(1, 461)	^*^
sentence_dur_cent	−17.151	6.4	−2.642	6.50(1, 76)	^*^
Vend_yes:Pend_yes	16.140	27.4	0.587	0.33(1, 33)	n.s.

Formula: RT ~ 1 + Vend ^*^ Pend + Vend_structure + Pend_structure + priming + sentence_duration_centred + (1 + sentence_duration_centred + Pend ^*^ Vend | subject) + (1 + Pend ^*^ Vend | item). Asterisks indicate significance levels of effects.

* *p < 0.05*;

^**^p < 0.01;

*** *p < 0.001*.

### 4.2. Eye-movements

**Figure 2:**
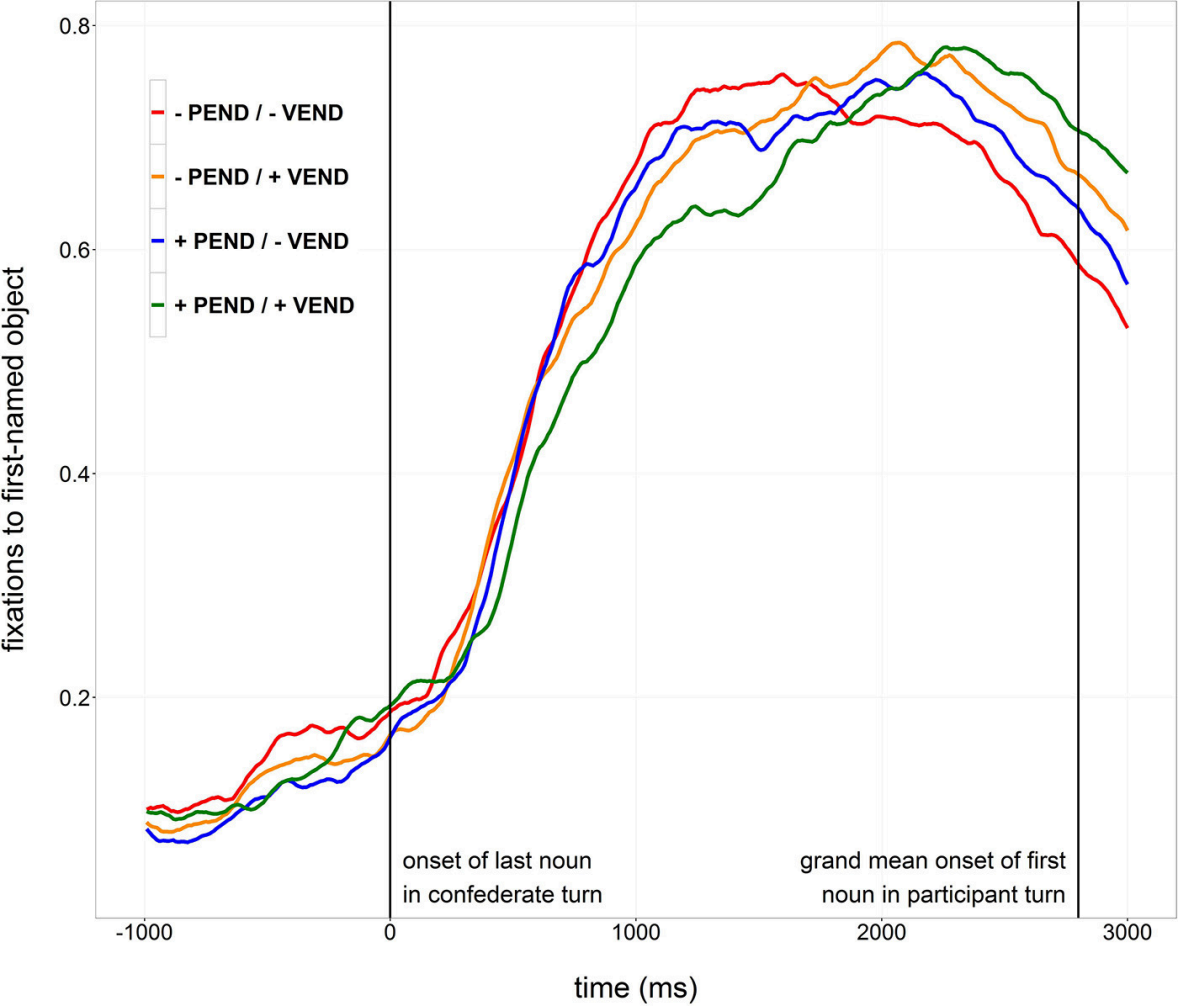
**Proportions of looks to the object named first by the participant, time-locked to the onset of the last object noun of the confederate turn (0 ms)**.

In order to explore the time course of participants' comprehension of the confederate's turn and the planning of their own response turn, fixations to the first-mentioned objects in the participants' responses were analyzed. Fixations toward an area of interest covering the first-named objects (target objects) and approximately 0.25 degrees of visual angle around them were categorized as target fixations. Figure 2 shows the proportions of target fixations time-locked to the beginning of the last noun in the confederate's utterance. Figure S1 shows proportions of looks to target objects time-locked to the offset of the incoming turn.

Participants' eye-movements were analyzed in a time window from 0 ms until 2800 ms, corresponding to the beginning of the last noun in the confederate's turn (0 ms) and the grand mean duration from the time-lock point until the beginning of the first object noun in the participant turn (2800 ms), respectively. Fixations to the target objects were aggregated to empirical logits in 100 ms time bins over the course of the analysis window by subjects and by items, respectively. This aggregation procedure removes non-independences in the eye movement data that arise from the way how eye movements are planned and executed (Barr, 2008). Where a participant looks at one point in time is highly dependent on where she was looking at the immediately preceding time point, as “[i]t is not physically possible for a participant's eye gaze to instantaneously travel from one region to another; the gaze must travel through time and space to reach its destination” (Barr, 2008, p. 464). Aggregating all observations from each participant or item for each condition into time bins and applying empirical logit transformations effectively accounts for the problem of non-independent observations. Only trials that included both looks for production and looks for comprehension were analyzed, excluding trials in which the confederate named none or all of the displayed objects. Ninety-two of the remaining trials were discarded due to trackloss, defined as missing data for a consecutive stretch longer than 500 ms within the time window of analysis. The final dataset included 2124 trials.

Visual inspection of the proportion of fixations indicates that target fixations started to slowly increase about half a second before the onset of the last object noun in the confederate's turn, probably because the set of candidate objects that needed to be named got smaller as the incoming turn unfolded (see Figure 2). The increase of fixations accelerated at about 400 ms after the onset of the last object noun in all conditions, meaning that participants moved their gaze for planning at about the same time for all turns, irrespective of their syntactic structure. From that point in time, it seems that fixations increased and decreased faster in conditions with a sentence-final verb than in items without a sentence-final verb. In conditions without a sentence-final verb, fixations appear to develop for the most part in parallel, irrespective of the projectability of the turn's ending. In conditions with a sentence-final verb however, fixations seem to differ from one another dependent on the projectability of the sentence final verb. In the condition with non-projectable sentence-final verbs (−Pend/+Vend), proportions appear to increase and decrease faster than in the condition with projectable sentence-final verbs (+Pend/+Vend).

Two pairs of conditions were compared to test for effects of verb position: trials with a projectable turn ending that contained a final verb were compared with trials with a projectable turn ending that did not contain a final verb (+Pend/+Vend vs. +Pend/−Vend, i.e., *kann…besorgen* vs. *sehe*); and trials with a non-projectable turn ending that contained a final verb were compared with trials with a non-projectable turn ending that did not contain a final verb (−Pend/+Vend vs. −Pend/−Vend, i.e., *habe…besorgt* vs. *habe*). Similarly, two pairs of conditions were compared to test for effects of projectability: trials that projectably ended in a turn-final verb were compared with trials that non-projectably ended a in turn-final verb (+Pend/+Vend vs. −Pend/+Vend, i.e., *kann…besorgen* vs. *habe…besorgt*); and trials that projectably ended after the last object noun were compared with trials that non-projectably ended after the last object noun (+Pend/−Vend vs. −Pend/−Vend, i.e., *sehe* vs. *habe*).

In each test, the interactions of Condition with the cubic time term (Time^3^) and the quadratic time term (Time^2^) were of most theoretical interest, as they model the hypotheses about the latency and speed of the increases of proportions of target looks in the different conditions. The linear time term (Time) itself does not directly relate to the hypotheses, as it only models a linear trend in increases of the proportions of target looks, which was expected to occur in all conditions as the task to name the remaining objects required participants to look at the target object in all conditions. An interaction between Condition and Time^3^ would indicate a difference in the latency of the increase of target fixations between conditions. An interaction of Condition and Time^2^ would indicate a difference in the steepness of the increase of target fixations between conditions. Table 4 shows an overview of the interactions in question and their statistical significance and Tables S1–S8 show summaries of the models and respective *F*-tests.

**Table 4:** **Eye-movement results of by-subject analysis**.

**Comparison**	**Effect**	**β**	**SE**	***F***	**sig**.
−Pend/-Vend vs.	t^2^ × cond.	0.52	0.23	*F*_(1, 727)_ = 4.64	^*^
−Pend/+Vend	t^3^ × cond.	−0.06	0.24	*F*_(1, 721)_ = 0.06	n.s.
+Pend/-Vend vs.	t^2^ × cond.	0.93	0.23	*F*_(1, 735)_ = 15.21	^***^
+Pend/+Vend	t^3^ × cond.	−0.37	0.28	*F*_(1, 393)_ = 1.55	n.s.
−Pend/-Vend vs.	t^2^ × cond.	0.32	0.25	*F*_(1, 651)_ = 1.54	n.s.
+Pend/-Vend	t^3^ × cond.	0.06	0.26	*F*_(1, 554)_ = 0.05	n.s.
−Pend/+Vend vs.	t^2^ × cond.	0.71	0.21	*F*_(1, 869)_ = 10.89	^***^
+Pend/+Vend	t^3^ × cond.	−0.23	0.23	*F*_(1, 843)_ = 1.00	n.s.

*Pairwise comparisons of Time^2^ × Condition and Time^3^ × Condition effects in growth curve analyses. t^2^ = Time^2^, t^3^ = Time^3^. Asterisks indicate significance levels of effects*.

* *p < 0.05*;

^**^p < 0.01;

*** *p < 0.001*.

*By-item analysis yielded similar pattern of results (see Tables S2, S4, S6, S8)*.

Throughout the pairwise comparisons, no interaction effect of Condition × Time^3^ reached statistical significance, with the single exception of the by-item comparison of +Pend/−Vend trials vs. +Pend/+Vend trials, indicating that the proportions of target looks started to increase at the same point in time in all conditions.

All four comparisons testing for the effects of verb position showed a statistically significant interaction of Condition × Time^2^, indicating steeper increases and decreases of target fixations in trials without sentence-final verbs as compared to trials with sentence-final verbs, irrespective of whether the turns' endings were projectable or not.

Neither the by-participant, nor the by-item comparison of −Pend/−Vend trials with +Pend/−Vend trials showed an interaction of Condition × Time^2^, meaning that target fixations increased in the same way in trials without a final verb form, no matter whether the turns' endings were projectable or not. However, both the by-participant and the by-item comparison of −Pend/+Vend trials with +Pend/+Vend trials showed an interaction of Condition × Time^2^ indicating that target fixations increased more slowly when the final verb was projectable than when it was not.

Because the finding that participants started gazing at the target object at the same time in all four conditions is based on null effects in the growth curve analyses, breakpoint analyses were conducted for each condition (Baayen, 2008) in order to ensure that the proportions of target looks did indeed start to increase at the same point in time in all conditions. Breakpoint analysis is based on regression modeling and seeks to identify discontinuities in linear relations, i.e., changes in slope. To identify when participants started to fixate on the target object, a search for breakpoints in target fixations was conducted in a time window between 200 ms after the onset of the last noun in the confederate turn and the grand mean beginning of the participant turn (900 ms) in steps of 100 ms. In the by-participant analyses, breakpoints were located around 400 ms after the onset of the last object noun for all conditions. The by-item analyses yielded a similar pattern of results (−Pend/−Vend: 500 ms, −Pend/+Vend: 400 ms, +Pend/−Vend: 400 ms, +Pend/+Vend: 300 ms, all conditions together: 400 ms). These results confirm that, irrespective of the incoming turn's structure, participants moved their gaze toward the target object as soon as the last object noun became recognizable, assuming that planning and executing a saccade in response to a linguistic stimulus takes about 200 ms (Allopenna et al., 1998).

## 5. Discussion

This study investigated how speakers coordinate listening and speech planning in a dialogue situation. We contrasted two hypotheses: The Late Planning Hypothesis, as formulated by Sjerps and Meyer (2015), stating that next speakers start planning their response only at the end of the incoming turn, and the Early Planning Hypothesis, as included in the turn-taking model of Levinson and Torreira (2015), stating that next speakers start planning as soon as all information that is needed to know what to respond is available. Furthermore, we investigated whether the timing of response planning relies on a projection of the incoming turn's completion point. Again, we contrasted two hypotheses: The Projection-Dependent Hypothesis, as formulated by de Ruiter et al. (2006), stating that next speakers depend on an accurate projection of the incoming turn's completion point to be able to begin planning their response, and the Projection-Independent Hypothesis, as proposed by Levinson and Torreira (2015), stating that planning can begin without an accurate projection of when the incoming turn will end.

To evaluate these hypotheses, an experiment was conducted that made use of the list-completion paradigm, a novel turn-taking paradigm that included two interlocutors, a confederate and a naive participant. The two participants engaged in a cooperative dialogue task that included naming objects on their screens. Which objects participants had to name depended on which objects were previously named by the confederate. Their conversation was recorded for an analysis of turn transition times and the participants' eye-movements were recorded for analyses of their gazes for comprehension and gazes for response planning.

Notably, the list-completion paradigm used both live and pre-recorded speech and thereby created a natural dialogue situation while at the same time allowing for tight control of critical utterances. The production task was highly naturalistic and resembled a conversational situation, as participants were not restricted to use a limited set of syntactic structures in their responses. The timing of responses was the same for pre-recorded sentences and sentences produced live. The data collected in this study can therefore be regarded as comparable to live situations, especially with respect to the fact that participants that whose data was included in the analysis stated that they did not notice the presence of pre-recorded material.

Participants were found to start planning their responses as soon as they knew which objects they had to name, gazing toward the objects they named in their responses as soon as the last object noun of the incoming turns could be recognized. As a consequence, they spent more time planning during the incoming turn when it contained a turn-final verb than when it ended with the last object noun, which led to faster responses after turns with a turn-final verb compared to turns without a turn-final verb. These results support the Early Planning Hypothesis and are in line with the model by Levinson and Torreira (2015) and with the findings of Bögels et al. (2015b), who found that when participants had to answer quiz questions, they started planning their responses as soon as the questions could be understood, no matter if that point in time was in the middle or at the end of the question. They are also in line with the findings by Magyari et al. (under review), who found that participants reacted faster to questions about objects on the screen when the answers to the questions could be known longer before the ends of the questions. This advantage of early planning may be an important factor in keeping inter-turn gaps short in conversation.

On the other hand, the results appear to be at odds with the results obtained by Sjerps and Meyer (2015), who found that participants did not start planning until right before or at the end of the incoming turn when taking turns with a computer in naming rows of four pictures. In that study, participants could, in principle, have begun to plan their utterance as soon as they had identified the first noun of the incoming turn, but were found to initiate planning only when they had heard the final noun. In both the present study and the study by Sjerps and Meyer, the measurement of utterance planning was time-locked to the last noun of the incoming turn. In Sjerps and Meyer's study, utterance planning could have been initiated much earlier but apparently participants opted for a late planning strategy. In contrast, in the present study, planning could not have been initiated any earlier but it could have been initiated later in cases where the incoming turn ended in a verb. However, participants apparently opted for an early planning strategy.

In these two studies, participants were in different communicative situations. While in Sjerps and Meyer's study no human interlocutor was present, in the present study participants interacted with another person in a joint task, which might have encouraged them to plan their utterances as soon as all relevant information was available rather than awaiting the end of the turn. Another difference lies in the structures of the utterances heard and produced. Conceptually and linguistically, the task used by Sjerps and Meyer was undoubtedly easier and more constrained than the task used in the present study. Given the simple nature of the planning task in the study by Sjerps and Meyer, participants could afford to postpone utterance planning until the preceding turn was completed. It seems that if next speakers consider the gain of early planning to be low, they can opt for late planning, as in Sjerps and Meyer's study. If, however, next speakers are under pressure to respond in a timely fashion, as they are in a conversational setting (Sacks et al., 1974), they can opt for early planning, resources permitting. The latter situation is arguably more frequent in everyday conversation, where planning might even start based on an anticipation of the incoming turn's message in order to keep inter-turn gaps short. The onset of planning might therefore depend on the information density at the end of the incoming turn (Jaeger, 2006, 2010), which was much higher in the present study than in the study by Sjerps and Meyer. In the present study, the incoming turn contained task-relevant information either until the last word, when the incoming turn ended in a noun, or until the last but one word, when a turn final verb was present. In the sentences used in the study by Sjerps and Meyer, on the other hand, only the first of four nouns was critical for the task, so that the last nine words of each presented sentence were irrelevant for the participants to follow their instructions.

While participants were found to start planning their responses before the end of the incoming turns, this planning during incoming speech was associated with additional processing costs. This conclusion results from two findings. First, proportions of looks for planning increased faster in turns not containing a sentence-final verb than in turns that ended in a verb. And second, even though response planning was already initiated before a sentence-final verb would be heard, response latencies after verb-final turns were shorter than after turns without a final verb by only a fraction of the length of the sentence-final verb. The reduction of the difference in response latencies might, at least partly, arise from interference of the turn-final material with response planning, rendering planning less efficient during turn-final verbs than during silence. When planning during the incoming turn, next speakers still needed to parse the input and predict or detect the upcoming completion point. When planning in silence, there was no such extra effort, making response planning more efficient.

The projectability of the incoming turn's completion point, which was manipulated by using different verbs in second position [ambiguous *habe* (“have”), unambiguous *sehe* (“see”) or *kann* (“can”)], did not modulate response latencies, which supports the Projection-Independent Hypothesis, as predicted from the model by Levinson and Torreira (2015). The results illustrate that response planning can be initiated without an exact projection of further upcoming material or of the exact locus of the turn end. However, the conjunction *und* (“and”) or *nur* (“only”) preceded the final noun in all of the confederate's utterances, giving a cue that the turn would end after either one or two additional words. Thus, coarse projection of the turn-completion point was always possible. However, accurate projection of the turn-completion point was found to be unnecessary for response planning.

However, projectability was found to influence looking behavior when sentences contained turn-final verbs. The influence was in the opposite direction as expected, with the proportion of looks for planning increasing more slowly in turns where a final verb was projectable than in turns in which the final verb was not projectable. This difference in looking behavior did not lead to a difference in response latencies, however, and therefore cannot be interpreted as a difference in planning difficulty. It could rather be a manifestation of a specific planning strategy, as participants seemed to distribute their planning effort more evenly over time when they were presented with turns that projectably allowed them to take extra time for planning at the end of the incoming turn. They may have done this by planning their response early conceptually, returning their gaze for comprehension, and finally looking for planning again to formulate and articulate the target object's name. With such a strategy, next speakers could have avoided inefficiencies in planning due to interference of incoming speech and thereby reduced cognitive effort.

Taken together, the results suggest that the timeline of the processes involved in taking turns in a conversation is far from ballistic. Contrary to classical monologic tasks commonly used in psycholinguistic studies, conversational situations are more complex and allow for more variability in the succession of the different aspects of language processing, especially regarding the interplay of comprehension and production. Cognitive resources seem to be distributed depending on the needs and possibilities of different conversational situations and may well be influenced by interlocutors' decisions and preferences. Since conversation can be regarded as the core ecology of language, this variability deserves more attention in future psycholinguistic research, calling for further studies concerning the psychology of dialogue in order to understand (the limits of) the involved flexibility, which is responsible for the general tendencies in turn-taking behavior as well as the observable deviations from them.

## 6. Conclusion

In this experiment, participants started to plan their responses as early as possible. Starting to plan a response during the incoming turn is costly, but leads to efficient timing of turn-taking and might be a key factor to keep gaps between turns in conversation short. Early planning does not depend on accurate projection of the incoming turn's completion point. The results support turn-taking models that include early response planning (Sacks et al., 1974; Heldner and Edlund, 2010; Levinson, 2012; Levinson and Torreira, 2015).

## Ethics statement

The experiment was approved by the Ethics Committee of the Faculty of Social Sciences, Radboud University Nijmegen. Participants signed a consent form prior to the study.

## Author contributions

MB, SL, and AM designed the experiment; MB conducted the experiment; MB and SS analyzed the data; MB wrote the paper and SS, SL, and AM commented on it.

## Funding

This research was financed by the ERC Advanced grant # 269484 INTERACT awarded to SL and by the Max Planck Institute for Psycholinguistics.

### Conflict of interest statement

The authors declare that the research was conducted in the absence of any commercial or financial relationships that could be construed as a potential conflict of interest.
