# Supplementary material for: The Timing of Utterance Planning in Task-Oriented Dialogue: Evidence from a Novel List-Completion Paradigm
Source: Front Psychol. 2016 Dec 1;7:1858. doi: 10.3389/fpsyg.2016.01858 (PMC5131015; doi:10.3389/fpsyg.2016.01858)
Supplement: Supplementary file 1 [file Image1.pdf]

Supplementary Material: The timing of utterance planning in task-oriented dialogue

Mathias Barthel, Sebastian Sauppe, Stephen C. Levinson, and Antje S. Meyer
*Correspondence:
Mathias Barthel
mathias.barthel@mpi.nl

1 SUPPLEMENTARY TABLES AND FIGURES

1.1 Eye-movements time-locked to the end of turn 1

As can be seen in Figure S1, proportions of target looks start to increase earlier in +Vend conditions than in -Vend conditions, namely one second versus about half a second before the offset of the incoming turn, respectively. Proportions of target looks in -Vend conditions seem to develop in parallel. In +Vend items however, proportions seem to differ from one another. In items with a non-projectable sentence-final verb (-Pend/+Vend), the increase of proportions appears to be steeper than in items with a projectable sentence-final verb (+Pend/+Vend).

1.2 Eye-movement statistics

Formula for all comparisons: emplogit $\sim$ 1 + (time + time^2 + time^3) * condition + (1 + (time + time^2 + time^3) * condition | subject) or: emplogit $\sim$ 1 + (time + time^2 + time^3) * condition + (1 + (time + time^2 + time^3) * condition | item), respectively. Asterisks indicate significance levels of effects. * p <.05; ** p <.01; *** p <.001

	Estimate	SE	*t*	*F*(Df,Df.res)	sig.
(Intercept)	0.461	0.04	9.284		
time	3.289	0.29	11.059	108.21(1,98)	***
time^2	-2.618	0.22	-11.735	139.36(1,180)	***
time^3	0.361	0.12	2.971	8.10(1,501)	**
condition	0.054	0.09	0.589	0.05(1,175)	n.s.
time:condition	0.835	0.28	2.901	7.70(1,411)	**
time^2:cond	0.529	0.23	2.229	4.64(1,727)	*
time^3:cond	-0.066	0.24	-0.269	0.06(1,721)	n.s.

Table S1. Growth curve model and *F*-tests comparing -Pend/-Vend with -Pend/+Vend by-subject.

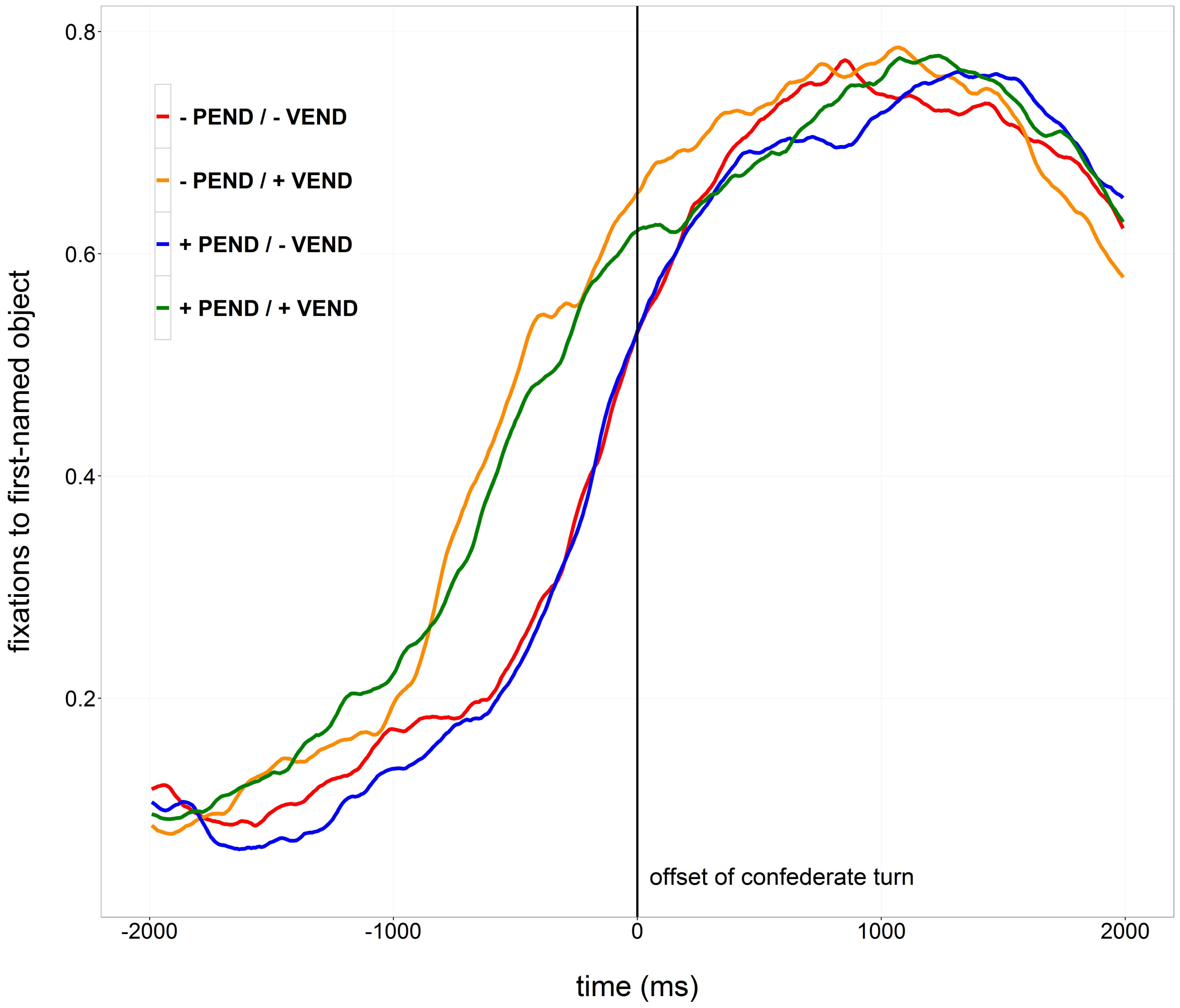

Figure S1. Proportions of looks to the target object time-locked to the offset of the confederate turn (0 ms).

	Estimate	SE	*t*	*F*(Df,Df.res)	sig.
(Intercept)	0.546	0.08	6.648		
time	3.532	0.22	15.976	260.33(1,189)	***
$time^2$	-2.443	0.17	-14.046	177.90(1,242)	***
$time^3$	0.256	0.14	1.720	2.81(1,306)	.
condition	-0.049	0.06	-0.756	1.73(1,506)	n.s.
time:condition	0.614	0.32	1.880	3.30(1,259)	.
$time^2$:cond	0.646	0.26	2.467	5.75(1,435)	*
$time^3$:cond	0.021	0.19	0.114	0.01(1,868)	n.s.

Table S2. Growth curve model and *F*-tests comparing -Pend/-Vend with -Pend/+Vend by-item.

	Estimate	SE	t	F(Df,Df.res)	sig.
(Intercept)	0.406	0.05	6.778		
time	3.709	0.31	11.845	129.54(1,89)	***
time^2	-2.07	0.23	-8.790	63.97(1,137)	***
time^3	0.274	0.15	1.819	2.77(1,440)	.
condition	-0.046	0.07	-0.666	0.03(1,437)	n.s.
time:condition	0.547	0.27	1.965	3.53(1,383)	.
time^2:cond	0.932	0.23	4.034	15.21(1,735)	***
time^3:cond	-0.374	0.28	-1.306	1.55(1,393)	n.s.

Table S3. Growth curve model and F-tests comparing +Pend/-Vend with +Pend/+Vend by-subject.

	Estimate	SE	t	F(Df,Df.res)	sig.
(Intercept)	0.517	0.08	5.813		
time	3.978	0.22	17.332	279.04(1,203)	***
time^2	-1.928	0.19	-9.753	93.27(1,187)	***
time^3	0.273	0.12	2.117	4.41(1,483)	*
condition	-0.019	0.06	-0.292	0.03(1,336)	n.s.
time:condition	0.937	0.32	2.857	7.68(1,269)	**
time^2:cond	0.845	0.30	2.809	7.45(1,350)	**
time^3:cond	-0.686	0.25	-2.661	6.72(1,465)	**

Table S4. Growth curve model and F-tests comparing +Pend/-Vend with +Pend/+Vend by-item.

	Estimate	SE	t	F(Df,Df.res)	sig.
(Intercept)	0.431	0.04	8.889		
time	3.146	0.32	9.704	72.45(1,90)	***
time^2	-2.707	0.21	-12.637	153.39(1,197)	***
time^3	0.422	0.14	2.884	7.65(1,451)	**
condition	-0.003	0.07	-0.047	0.34(1,298)	n.s.
time:condition	0.564	0.24	2.333	5.06(1,683)	*
time^2:cond	0.328	0.25	1.290	1.54(1,651)	n.s.
time^3:cond	0.061	0.26	0.234	0.05(1,554)	n.s.

Table S5. Growth curve model and F-tests comparing -Pend/-Vend with +Pend/-Vend by-subject.

	Estimate	SE	t	F(Df,Df.res)	sig.
(Intercept)	0.546	0.08	6.512		
time	3.363	0.21	15.983	260.06(1,205)	***
time^2	-2.553	0.19	-13.010	156.80(1,186)	***
time^3	0.427	0.13	3.162	9.65(1,408)	**
condition	-0.053	0.06	-0.809	0.09(1,295)	n.s.
time:condition	0.226	0.34	0.651	0.39(1,260)	n.s.
time^2:cond	0.390	0.26	1.462	2.01(1,384)	n.s.
time^3:cond	0.365	0.23	1.565	2.32(1,481)	n.s.

Table S6. Growth curve model and F-tests comparing -Pend/-Vend with +Pend/-Vend by-item.

	Estimate	SE	t	F(Df,Df.res)	sig.
(Intercept)	0.434	0.06	6.738		
time	3.836	0.28	13.544	159.23(1,105)	***
$time^2$	-1.991	0.24	-8.199	55.84(1,127)	***
$time^3$	0.205	0.14	1.385	2.35(1,444)	n.s.
condition	-0.108	0.07	-1.465	0.55(1,462)	n.s.
time:condition	0.265	0.31	0.846	0.64(1,301)	n.s.
$time^2$:cond	0.719	0.21	3.402	10.89(1,869)	**
$time^3$:cond	-0.237	0.23	-1.034	1.00(1,843)	n.s.

Table S7. Growth curve model and F-tests comparing -Pend/+Vend with +Pend/+Vend by-subject.

	Estimate	SE	t	F(Df,Df.res)	sig.
(Intercept)	0.511	0.08	6.145		
time	4.107	0.23	17.601	291.96(1,181)	***
$time^2$	-1.826	0.18	-9.954	111.99(1,241)	***
$time^3$	0.083	0.14	0.598	0.45(1,391)	n.s.
condition	-0.020	0.07	-0.271	1.02(1,356)	n.s.
time:condition	0.557	0.29	1.906	3.41(1,314)	.
$time^2$:cond	0.628	0.28	2.245	4.76(1,398)	*
$time^3$:cond	-0.335	0.21	-1.525	2.21(1,556)	n.s.

Table S8. Growth curve model and F-tests comparing -Pend/+Vend with +Pend/+Vend by-item.
